# Incorporation of machine learning and deep neural network approaches into a remote sensing-integrated crop model for the simulation of rice growth

**DOI:** 10.1038/s41598-022-13232-y

**Published:** 2022-05-30

**Authors:** Seungtaek Jeong, Jonghan Ko, Taehwan Shin, Jong-min Yeom

**Affiliations:** 1 Korea Aerospace Research Institute, 169-84 Gwahak-ro, Yuseong-gu, Daejeon, 34133 Republic of Korea; 2 Applied Plant Science, Chonnam National University, 77 Yongbong-ro, Buk-gu, Gwangju, 61186 Republic of Korea

**Keywords:** Computational biophysics, Ecology, Agroecology, Ecological modelling, Plant sciences, Plant ecology

## Abstract

Machine learning (ML) and deep neural network (DNN) techniques are promising tools. These can advance mathematical crop modelling methodologies that can integrate these schemes into a process-based crop model capable of reproducing or simulating crop growth. In this study, an innovative hybrid approach for estimating the leaf area index (LAI) of paddy rice using climate data was developed using ML and DNN regression methodologies. First, we investigated suitable ML regressors to explore the LAI estimation of rice based on the relationship between the LAI and three climate factors in two administrative rice-growing regions of South Korea. We found that of the 10 ML regressors explored, the random forest regressor was the most effective LAI estimator, and it even outperformed the DNN regressor, with model efficiencies of 0.88 in Cheorwon and 0.82 in Paju. In addition, we demonstrated that it would be feasible to simulate the LAI using climate factors based on the integration of the ML and DNN regressors in a process-based crop model. Therefore, we assume that the advancements presented in this study can enhance crop growth and productivity monitoring practices by incorporating a crop model with ML and DNN plans.

## Introduction

Process-based crop models can simulate sequential variations in crop growth as a function of mathematical procedures^1,2^. Although these crop models deliver a reliable simulation performance, assembling the different spatial inputs and complicated crop parameters can substantially restrict the modeling efficiency^3^. Despite spatiotemporal limitations in observation, remote sensing (RS) can be a valuable technique for observing dynamic spatial variations in crop growth and development within plant ecosystem environments, depending on RS platforms^4^. A hybrid approach, combining a crop model with RS information, may increase the advantages of both and compensate for the weaknesses of the individual techniques, filling the spatiotemporal gaps in both RS and simulation data^5,6^. Therefore, there have been extensive efforts to advance crop simulation performances by incorporating RS information using various data assimilation approaches involving RS and crop modelling^6–8^. For instance, the RS-integrated crop model (RSCM) is based on a hybrid scheme and is used to simulate staple crops, including barley, paddy rice, soybean, and wheat^6,9–11^. RSCM can incorporate the leaf area index (LAI) or vegetation indices (VIs) from various types of RS data.

LAI has been employed as a critical variable for simulating sequential crop growth in most process-based crop models integrated with RS data and RSCM as a function of mathematical optimisation procedures^7,8^. The LAI variable in these crop models is formulated using the linear relationship with VIs obtained from various RS platforms^6,12^. However, developing steady mathematical formulations presents some challenges, including the dimensional (D) differences between LAI (that is, 3-D) and VIs (that is, 2-D), possible variations in the relationship among different RS platforms, and dynamic relational variabilities among other crop species that are even apparent in different growth stages, specifically during leaf senesce. Therefore, a novel approach for a consistent LAI estimation and improving the performance of the process-based crop models incorporated with RS data, including RSCM, should be investigated.

Deep neural network (DNN) and machine learning (ML) techniques are promising tools for advancing mathematical crop modelling methodologies to integrate these schemes into a process-based crop model capable of reproducing and predicting crop growth and development. ML has proven to be an effective method for addressing the limitations of conventional empirical methods in the simulation of crop yield using RS data because it considers nonlinearity between the input variables and crop yield^13–15^. Therefore, some efforts have been made to incorporate an ML approach with a crop model to advance yield estimation^16–18^. These study approaches included simulation crop model variables as input features in ML models. Certain reports have also revealed that recent improvements in DNN methodologies, based on their powerful prediction performance, are applicable to the more advanced and precise simulation of crop yields^19,20^. The ML and DNN methodologies applied to crop yield prediction encompass, but are not limited to, the support vector machine, random forest (RF), dimensional convolutional neural network, and long short-term memory. Popular DNN applications in agriculture include weed identification, land cover classification, plant recognition, fruit counting, and crop type classification^21^. Therefore, it appears that the ML and DNN approaches have been adopted to address each attribute of crop productivity and management, which is being correlated with its biotic and abiotic environments.

We assume that ML and DNN methodologies can improve the simulation performances of present mathematical crop models by effectively assimilating these data-driven modelling techniques. While some earlier hybrid efforts were to add simulation crop model variables to ML models, integrating ML or DNN processes into a mathematical crop model has not been researched. Therefore, in this study, we aimed to develop an innovative hybrid approach of integrating ML and DNN methodologies into a process-based crop model for estimating the LAI of rice. We investigated suitable ML and DNN models to calculate the LAI values of rice (*Oryza sativa*) based on the relationship between LAI and weather factors.

## Results

**Figure 1:**
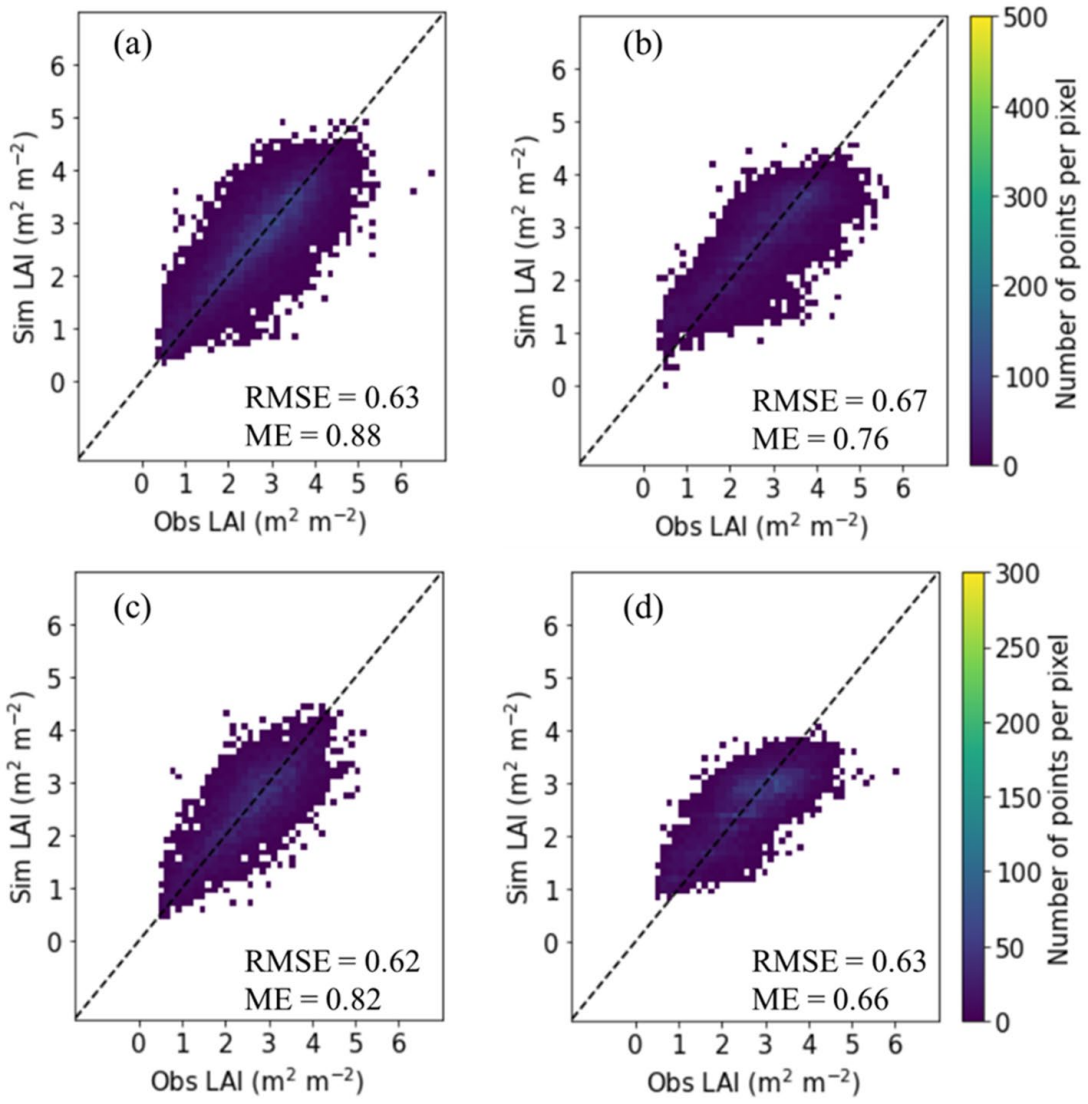
Simulated (Sim) versus observed (Obs) leaf area index (LAI) of paddy rice using the (**a** and **c**) random forest (RF) regressor and (**b** and **d**) deep neural network (DNN) regressor in (and **b**) Cheorwon (n = 10,388) and (**c** and **d**) Paju (6,622), South Korea. RMSE and ME stand for root mean square error and model efficiency.

Training scores using 10 machine learning regressors for the regression analyses of LAI with respect to three climate factors for rice in Cheorwon ranged from 0.441 to 0.863, whereas test scores varied from 0.428 to 0.622 (Table 1). Training scores for those in Paju ranged from 0.423 to 0.855, whereas test scores varied from 0.416 to 0.568. Assuming that the RF regressor was the best working model in both regions based on the test scores (that is, 0.622 in Cheorwon and 0.568 in Paju), we analysed its capabilities for simulating LAI compared to that of the DNN regressor. In Cheorwon, simulated LAI values agreed with the corresponding observed LAI values with a root mean square error (RMSE) of 0.63 m^2^ m^−2^ and a normalised Nash–Sutcliffe model efficiency (ME) of 0.88 using the RF regression (Fig. 1a) and an RMSE of 0.67 m^2^ m^−2^ and an ME of 0.76 using the DNN regression (Fig. 1b). In Paju, simulated LAI values agreed with the observed LAI values with an RMSE and ME of 0.62 m^2^ m^−2^ and of 0.82, respectively, for the RF regression (Fig. 1c) and an RMSE and ME of 0.63 m^2^ m^−2^ and 0.66, respectively for the DNN regression (Fig. 1d).

**Table 1:** Training and test scores for regression analyses of leaf area index (LAI) with respect to climate factors using 10 machine learning (ML) regressors for rice in Cheorwon and Paju, South Korea.

Regressor	Cheorwon		Paju	
	Training score	Test score	Training score	Test score
Polynomial linear	0.498	0.490	0.427	0.417
Ridge	0.498	0.490	0.427	0.417
Lasso	0.441	0.428	0.426	0.416
Support vector	0.513	0.500	0.475	0.459
Random forest	0.843	0.622	0.828	0.568
Extra trees	0.863	0.590	0.855	0.489
Gradient boosting	0.549	0.543	0.508	0.499
HGB	0.611	0.590	0.579	0.551
XGB	0.671	0.613	0.650	0.561
LightGBM	0.612	0.590	0.580	0.552

HGB, XGB, and LightGBM stand for Histogram-based Gradient Boosting, Extreme Gradient Boosting, and Light Gradient Boosting machine regression.

**Figure 2:**
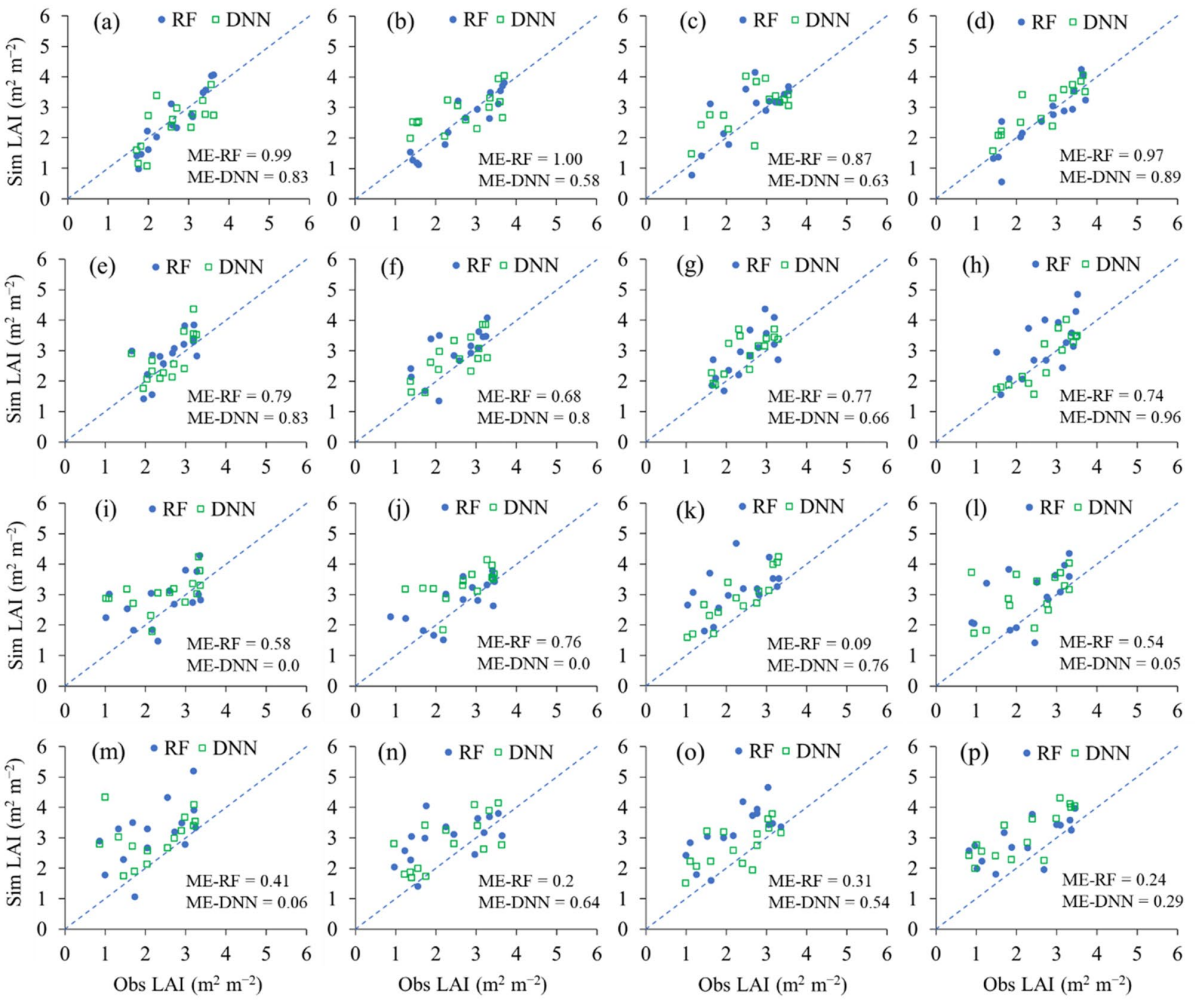
Simulated (Sim) versus observed (Obs) LAI of paddy rice using the RF and DNN regressors for (**a**–**d**) Cheorwon, (**e**–**h**) Paju, (**i**–**l**) Gimje, South Korea, and (**m**–**p**) Pyeongyang, North Korea in (**a**, **e**, **i**, and **m**) 2014, (**b**, **f**, **j**, and **n**) 2015, (**c**, **g**, **k**, and **o**) 2016, and (**d**, **h**, **l**, and **p**) 2017. ME-RF and ME-DNN represent the model efficiency of RF and the model efficiency of DNN.

We applied the RF and DNN regressors considering enhancement for reproducing the regional rice growth for Cheorwon, Paju, and Gimje, South Korea and Pyeongyang, North Korea from 2014 to 2017 (Fig. 2). The calibrated regression models were cross-validated between Cheorwon and Paju while those developed for Cheorwon were applied for the typical rice growing regions of Gimje, South Korea and Pyeongyang, North Korea. In Cheorwon and Paju, simulated LAI values corresponded to the observed LAI values, with an RMSE range of 0.34–0.81 m^2^ m^−2^ and an ME range between 0.68 and 0.1 using the RF regression and an RMSE range of 0.42–0.78 m^2^ m^−2^ and an ME range of 0.58–0.96 using the DNN regression (Table 2). In Gimje and Pyeongyang, simulated LAI values corresponded to the observed LAI values with an RMSE range of 0.63–1.18 m^2^ m^−2^ and an ME range of 0.09–0.76 using the RF regression and an RMSE range of 0.72–1.1 m^2^ m^−2^ and an ME range of 0.0–0.76 using the DNN regression.

**Table 2:** Comparison of observed (Obs) and simulated (Sim) LAI values of paddy rice in terms of the root mean square error (RMSE) and Nash–Sutcliffe efficiency (NSE) for the random forest (RF) and deep neural network (DNN) regressors for Cheorwon (CW), Paju (PJ), Gimje (GJ), South Korea and Pyeongyang (PY), North Korea, from 2014 to 2017.

Site	Year	RF				DNN			
		Sim	Obs	RMSE	ME	Sim	Obs	RMSE	ME
		m^2^ m^−2^			None	m^2^ m^−2^			None
CW	2014	2.63	2.55	0.38	0.99	2.63	2.48	0.58	0.83
	2015	2.66	2.56	0.34	1.00	2.66	2.87	0.67	0.58
	2016	2.61	2.89	0.64	0.87	2.61	2.99	0.78	0.63
	2017	2.66	2.62	0.46	0.97	2.66	2.97	0.49	0.89
PJ	2014	2.59	2.86	0.57	0.79	2.59	2.78	0.56	0.83
	2015	2.47	2.93	0.73	0.68	2.47	2.77	0.55	0.80
	2016	2.48	2.90	0.67	0.77	2.48	2.95	0.63	0.66
	2017	2.69	3.16	0.81	0.74	2.69	2.74	0.42	0.96
GJ	2014	2.45	2.82	0.84	0.58	2.45	3.04	0.92	0.00
	2015	2.56	2.79	0.63	0.76	2.68	3.34	0.89	0.00
	2016	2.25	3.16	1.18	0.09	2.25	2.85	0.72	0.76
	2017	2.34	2.96	1.02	0.54	2.34	2.99	1.04	0.05
PY	2014	2.19	3.14	1.25	0.41	2.19	2.99	1.18	0.06
	2015	2.29	2.99	1.05	0.20	2.29	2.82	0.88	0.64
	2016	2.25	3.23	1.14	0.31	2.25	2.73	0.77	0.54
	2017	2.17	2.89	0.99	0.24	2.17	3.12	1.10	0.29

**Figure 3:**
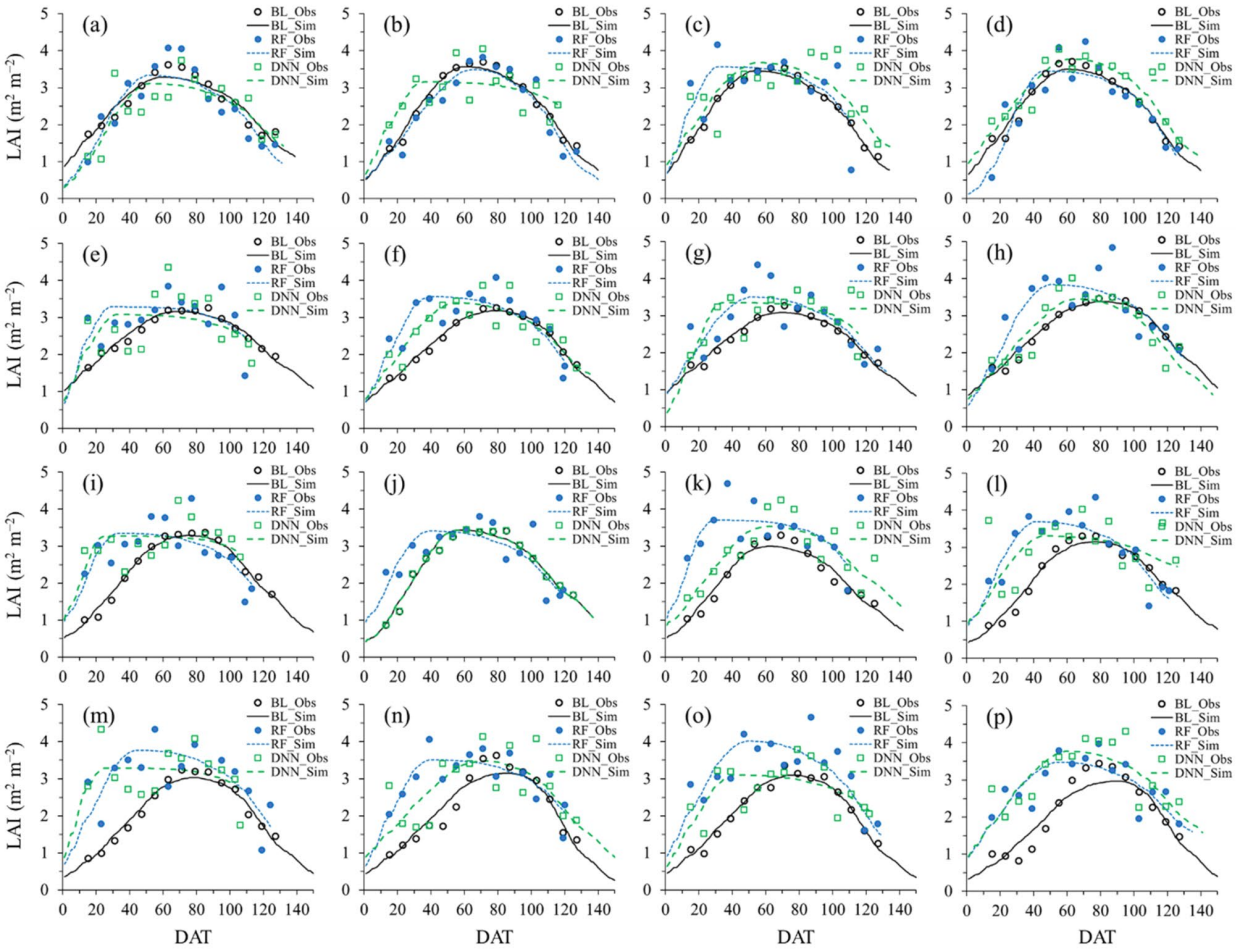
Simulated (Obs) versus observed (Obs) LAI of paddy rice for the datasets, obtained using the RF and DNN regressors in comparison with the baseline (BL) for (**a**–**d**) Cheorwon, (**e**–**h**) Paju, (**i**–**l**) Gimje, South Korea and (**m**–**p**) Pyeongyang, North Korea in (**a**, **e**, **i**, and **m**) 2014, (**b**, **f**, **j**, and **n**) 2015, (**c**, **g**, **k**, and **o**) 2016, and (**d**, **h**, **l**, and **p**) 2017. Sim LAI values were produced using a remote sensing-integrated crop model, while Obs LAI values of the BL were obtained from the MODIS imagery.

The RF and DNN-estimated LAI values were applied for simulating LAI values at Cheorwon (Fig. 3a–d) and Paju (Fig. 3e–h) with cross-validation. In addition, the RF and DNN-estimated LAI values using the Cheorwon dataset were applied at Gimje (Fig. 3i–l) and Pyeongyang (Fig. 3m–p) employing the RSCM regime. As a result, simulated LAI values more closely corresponded to the RF and DNN-estimated LAI values in the cross-validation at Cheorwon and Paju than those at Gimje and Pyeongyang. The most LAI disagreement values in the RSCM simulation with the BL values were observed during the early rice-growing season. The early season difference is attributed to the ML and DNN estimation inaccuracies.

## Discussion

This study adopted normalized differenc vegetation index (NDVI) and climate data from satellite and climate projection model data to reproduce the rice LAI and develop an integrated crop modelling approach through an ML or DNN technique. We employed this approach to obtain large datasets that allow effective ML and DNN modelling. We observed that the RF regressor was the best working model for simulating the rice LAI in the regions of interest; furthermore, it outperformed the DNN regressors. However, the finding of the current study conflicts with earlier research reports of DNN approaches outperforming state-of-the-art ML approaches^22,23^. Therefore, it appears that the simulation outcomes depend on the data scope and associated features. The dataset that we employed indicated the supremacy of ML approaches. However, it is possible that using more extensive data than those implemented in the present study or applying other latest DNN structures may produce results more in line with those of earlier research^22,23^, which exhibited the efficacy of DNN regressors.

Using satellite-based datasets in this study had the following respective advantages and disadvantages: reproducing the rice LAI and obtaining solar radiation but using the local climate projection model to produce temperatures. The advantages included the availability of big data and accessibility of the regions of interest, depending on the satellite paths. The disadvantages included limited spatial, temporal, and radiometric resolutions, likely due to using different satellite sensors. Satellite imagery contains multiple pixels that allow researchers to implement ML and DNN methodologies using big data. This is also true for the local climate production data. However, the use of satellite imagery with a coarse spatial resolution (for example, Geostationary Ocean Color Imager (GOCI) or Moderate Resolution Imaging Spectroradiometer (MODIS)) can result in discrepancies, as observed in a small part in the current study, owing to errors from mixed-pixel consequences. The errors include the underestimation of small paddy areas and overestimation of large paddy areas (particularly areas with considerably heterogeneous land cover)^24,25^. These errors are even more apparent when performing estimations based on the equivalence of paddy patches because small areas are often untraceable^26–28^. Irrespective of this inaccuracy, it is necessary to use coarse ground resolution images at a high temporal resolution for continuous and sequential land cover classification and monitoring of important crop-growth information over large regions.

The current study showed that simulated LAI values agreed with the RF and DNN-estimated LAI values in the cross-validation at Cheorwon and Paju more tightly than the RF and DNN-estimated LAI values using the Cheorwon dataset for the evaluations at Gimje and Pyeongyang. This inconsistency should be directly associated with the regional distances between the parameterisation and evaluation datasets. Therefore, we assume that the inconsistency is attributable to different rice-growing environments and genetic factors to affect leaf growth untrained in the ML and DNN models developed in the Cheorwon environment. This issue could be addressed using the adjacent region application methodology (likewise, the cross-validation between Paju and Cheorwon). Another approach would be using a more wide-ranging area dataset encompassing the most different environments and rice cultivars while it is not out of the current research scope.

Meanwhile, nearly all disagreements in the LAI estimates in the RSCM simulation were found during the early rice-growing seasons of all the regions of interest. Therefore, we assume some inconsistency outcomes could be related to biological or abiotic factors influencing early-season rice growth. For example, the lower observed LAI values might be attributed to reduced leaf growth due to environmental stresses such as drought or damage from microbial or insect occurrences caused by warmer weather conditions.

Integrating RS or satellite data into the process-based crop model (RSCM regime) offered several advantages. First, the model requires reasonably small input parameters and variables, in which existing observations are introduced as critical factors in the representation of environmental circumstances. Second, the method allows the RSCM regime to improve the simulation performance. Third, it enables RSCM to incorporate RS information from various operational optical satellite-based sensors of varying spatial resolutions^6,29,30^ and other platforms such as remotely controlled aerial systems^31^. Finally, the RSCM regime in the methodology is applicable to any region of interest on the Earth’s surface, including data-sparse and inaccessible regions^30,32^, as long as satellite images are attainable. The optimisation technique was designed to incorporate RS data from various platforms into the RSCM regime, causing it to closely rely on the LAI inputs established from the remotely sensed information. However, the RSCM optimisation methodology has several constraints, including the incomplete representations of RS information and restricted observations during the crop-growing season. These limitations can eventually cause inconsistencies between the simulations and observations and inaccurate predictions of crop growth and productivity.

In conclusion, this study validated the feasibility of integrating an ML or DNN approach into a process-based crop model that uses RS data. First, we investigated the modelling performances of available ML regression models to simulate paddy rice LAI using three climate factors. The test scores obtained to estimate the rice LAI using the 10 ML regression models indicated the best performance scores in both the regions of Cheorwon and Paju with the RF regressor. Furthermore, we noted that a well-calibrated state-of-the-art ML model, such as RF, could reproduce the rice LAI using climate factors at least as effective as a well-trained DNN regressor. Therefore, we propose that the innovation of integrating an ML or DNN scheme into a process-based crop model can improve crop growth and productivity monitoring methodologies. Although this paper proposes an innovative integration approach for RSCM with an ML regressor using climate data, further efforts are required to incorporate ML or DNN methodologies such as an advanced hybrid system employing the LAI and VIs relations.

## Methods

### Study locations and rice data

**Figure 4:**
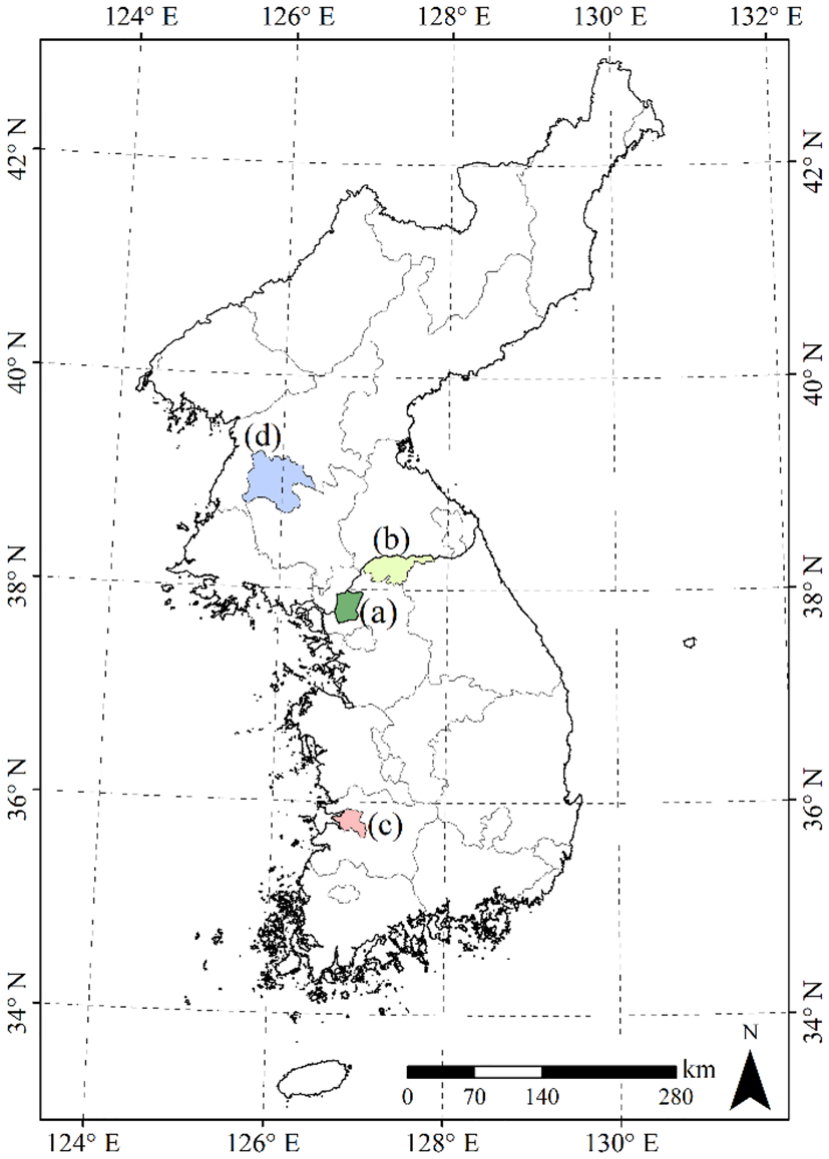
Study location boundary maps of (**a**) Cheorwon, (**b**) Paju, (**c**) Gimje in South Korea and (**d**) Pyeongyang in North Korea.

The ML and DNN models were developed for the rice growing areas in the entire geographic regions of Cheorwon and Paju in South Korea (Fig. 4). Then, the parameterised ML and DNN models were evaluated for the representative rice growing areas of Gimje, South Korea and Pyeongyang, North Korea. Cheorwon and Paju were selected as these areas are typical rice cultivation regions in the central portion of the Korean peninsula. The paddy rice cultivation regions in Cheorwon and Paju have areas of 10,169 and 6,625 ha, respectively, representing 80.4% and 62.6% of the total staple croplands for each region, according to the Korean Statistical Information Service, KOSIS (https://kosis.kr/).

The leading rice cultivar in Cheorwon and Paju was *Odae* (bred by NICS in 1983), cultivated in more than 80% of the paddy fields during the study period, according to KOSIS. Rice seedlings were transplanted in these areas between May 15 and 20, deemed as the ideal transplanting period.

### Cumulative crop NDVI data

We used the temporal profiles of NDVI from the Terra MODIS MOD09A1 surface reflectance 8-day product with a spatial resolution of 500 m, which were employed for the ML and DNN model input variable. This product is the composited imagery by selecting the best pixels considering the cloud and solar zenith during eight days^33^. It is essential to secure reliable and continuous phenological NDVI data for determining crop yield in monsoon regions like the current study area concerning input variables for the process-based crop model. Therefore, the cloud-contaminated pixels were interpolated with other poor quality pixels caused by aerosol quantity or cloud shadow using the spline interpolation algorithm during the rice-growing season to improve data quality during the monsoon season. This approach has been widely used in time series satellite imagery for interpolation^34–36^. The criteria for poor quality pixels for interpolation were determined from the 16-bit quality assurance (QA) flags from the MOD09A1 product^33^.

### Weather data

Furthermore, we estimated the incoming solar radiation on the surface (insolation) obtained from the COMS Meteorological Imager (MI). Insolation reflects the energy source of photosynthesis for the crop canopies. We adopted a physical model to estimate solar radiation by considering atmospheric effects such as aerosol, water vapour, ozone, and Rayleigh scattering^37–41^. Before estimating the solar radiation from the physical model, we classified clear and cloudy sky conditions because cloud effects should be considered for their high attenuation influences. If the pixel image was assigned as a clear sky condition, atmospheric parameterisations were performed for direct and diffuse irradiance owing to the effects of atmospheric constituents and solar-target-satellite sensor geometry^40,42–44^. If the pixel images were considered as under cloudy conditions, the cloud attenuation was calculated using a cloud factor for visible reflectance and the solar zenith angle^42^. Finally, the estimated solar radiation from COMS MI was used as one of the main input parameters of the RSCM system. Comprehensive descriptions of those parameters used for the physical model can be referenced from earlier studies^41,43^.

The maximum and minimum air temperature data were obtained from the Regional Data Assimilation and Prediction System (RDAPS) provided by the Korea Meteorological Administration (KMA, https://www.kma.go.kr). The spatial resolution of the RDAPS is 12 km, and it is composed of 70 vertical levels up to about 80 km. The global data assimilation and prediction system is provided at 3-h intervals for the Asian regions, and forecasts are performed four times a day (00, 06, 12, and 18 UTC) for 87 h. In addition, the system is operated in a 6-h interval analysis-prediction-circulation system using the four-dimensional variational data assimilation^45^. The weather datasets were resampled to a spatial resolution of 500 m using the nearest neighbour method that does not change the existing values to match the MODIS imagery.

### Process-based crop model

The current study employed the RSCM to incorporate an ML and DNN procedure and then simulate rice growths and yields (Supplementary Fig. S1). We integrated an ML and DNN regressor into the RSCM-rice system based on the investigation of the ML or DNN regressors described in the following subsection. The ML or DNN scheme was implemented to improve the mathematical regression approach for the RS-based VIs and LAI relationships, as described below.

RSCM is a process-based crop model designed to integrate remotely sensed data, allowing crop modellers to simulate and monitor potential crop growth^6^. This model can accept RS data as input to perform its within-season calibration procedure^5^, wherein the simulated LAI values are compared to the corresponding observed values. Four different parameters (that is, *L*_0_, *a*,* b*, and *c*) are utilised in the within-season procedure to define the crop-growth processes based on the optimisation of LAI using the POWELL procedure^46^. In addition, these parameters can be calibrated using the Bayesian method to obtain acceptable values with a prior distribution that was selected based on the estimates from earlier studies^6,47^. The current research project applied consistent initial conditions and parameters to calibrate the RSCM-rice system.

### ML and DNN models

The ML models investigated in this study were Polynomial regression, Ridge, Least Absolute Shrinkage and Selection Operator (LASSO), Support Vector Regression (SVR), RF, Extra Trees (ET), Gradient Boosting (GB), Histogram-based Gradient Boosting (HGB), Extreme Gradient Boosting (XGB), and Light Gradient Boosting machine regression (LightGB) regressors. These models are implemented in scikit-learn (https://scikit-learn.org/), while the DNN model (Supplementary Fig. S4) is implemented in Keras (https://keras.io/), which are achievable on Python (https://www.python.org/).

The Polynomial regression model is a particular regression model to overcome the limitations of simple linear regression by estimating the relationship with the N^th^ degree polynomial. The Ridge and Lasso additionally use l2-norm and l1-norm as constraints in the existing model. These characteristics of the models show better performance than the conventional linear regression, which uses the least-squares method to find appropriate weights and biases to reduce overfitting^48,49^.

The SVR allows the definition of the amount of allowable error and finds a hyperplane of higher dimensions to fit the data. The SVR is widely used for classification and numerical prediction and is less overfitting and easier to use than neural networks. However, it takes a long time to build an optimisation model, and it is difficult to interpret the results^50^.

The RF is an ensemble model that trains multiple decision tree models and aggregates its results. It has good generalisation and performance, is easy to tune parameters, and is less prone to overfitting. On the other hand, memory consumption is higher than in other ML models. Also, it is not easy to expect higher performance improvement even when the amount of training dataset increases. Extra trees increase randomness by randomly splitting each candidate feature in the tree, which can reduce bias and variance^51^. The difference from the RF is that ET does not use bootstrap sampling but uses the whole origin data when making decision trees. The GB belongs to the boosting series among the RF ensemble models, which combines weak learners to create strong learners with increased performance. Meanwhile, the GB training process is slow and not efficient in overfitting. There are HGB, XGB, and LightGB in the form of the GB that improve performance by increasing the training speed and reducing overfitting. The HGB speeds up the algorithm by grouping each decision tree with a histogram and reducing the number of features. The XGB improves learning speed through parallel processing and is equipped with functions necessary to improve performance compared to the GB, such as regularisation, tree pruning, and early stopping. The LightGBM significantly shortens the training time and decreases memory use by using a histogram-based algorithm without showing a significant difference in predictive performance compared to the XGBoost^52^.

The DNN increases the predictive power by increasing the hidden layer between the input and the output layers. Non-linear combinations between input variables are possible, feature weighting is performed automatically, and performance tends to increase as the amount of data increases. However, since it is difficult to interpret the meaning of the weights, there is a disadvantage in that the results are also difficult to interpret. In addition, when fewer training datasets are collected, the performance of the ML models mentioned above can be better^53^.

This study used satellite-based solar radiation and model-based maximum and minimum temperatures to estimate LAI values during the rice-growing seasons on the study sites (Cheorwon, Paju, Gimje, and Pyeongyang) for seven years (2011–2017), employing the ML and DNN regressors. We reproduced rice LAI values from the MODIS-based NDVI values using the empirical relationship between LAI and NDVI (Supplementary Fig. S2). Cheorwon and Paju datasets were used for the ML and DNN model development, while Gimje and Pyeongyang datasets were employed for the model evaluation. The target LAI variable data used for the model development showed characteristic seasonal and geographical variations (Supplementary Figs. S3 and S4). The model development datasets were divided into train and test sets with a 0.8 and 0.2 ratio using the scikit-learn procedure. All the ML and DNN regressors were trained and tested, obtaining appropriate hyperparameters. Alpha values for the Ridge and Lasso were determined as 0.1 and 0.01 based on a grid search approach with a possible range of values (Supplementary Fig. S5). The activation function employed for the DNN model was the rectified linear unit (ReLU), implementing six fully connected layers with a design of gradual increasing and decreasing units from 100 to 1,000 (Supplementary Fig. S6). The model was performed with a dropout rate of 0.17, the ‘adam’ optimizer at a learning rate of 0.001, 1,000 epochs, and a batch size of 100. The DNN hyperparameters were determined based on a grid search approach and a trial and error approach, seeking minimum and steady losses for each study region (Supplementary Fig. S7).

### Evaluation of the model performance

We analysed the performance of the ML (that is, RF) and DNN regimes using two statistical indices in Python (https://www.python.org), namely the RMSE and the ME^54^. This index denotes the comparative scale of the residual variance of simulated data compared to the observed data variance. Furthermore, ME can assess the agreement between the experimental and simulated data, showing how well these data fit through the 1:1 line in a scatter plot. The index value can vary from − ∞ to 1. We employed normalized ME for advanced interpretation, allowing for the ME measure in simulation estimation approaches used in model evaluation. Thus, ME = 1, 0, and − ∞ correspond to ME = 1, 0.5, and 0, respectively. Therefore, the model is considered reliable if the ME value is nearer to 1, whereas the simulated data are considered less dependable if the ME value is close to 0.

## Supplementary Information

Supplementary Information.
