# Supplementary material for: Incorporation of machine learning and deep neural network approaches into a remote sensing-integrated crop model for the simulation of rice growth
Source: Sci Rep. 2022 May 30;12:9030. doi: 10.1038/s41598-022-13232-y (PMC9151665; doi:10.1038/s41598-022-13232-y)
Supplement: Supplementary file 1 — Supplementary Information. [file 41598_2022_13232_MOESM1_ESM.docx]

**Supplementary Information**


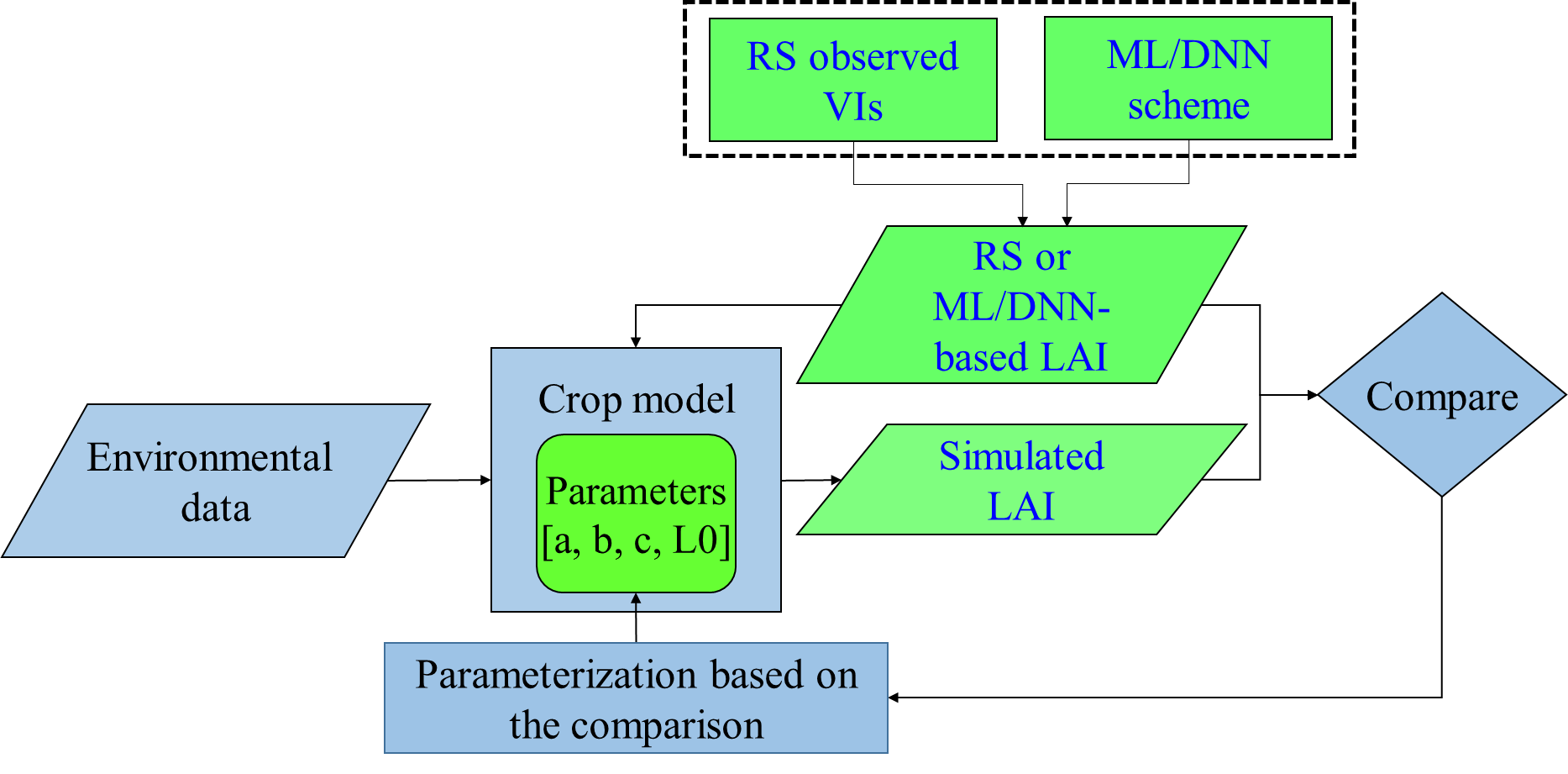


Supplementary Figure S1. Schematic diagram of the process-based crop model integrated with RS observed vegetation indices (VIs) or machine learning (ML) and DNN schemes to simulate LAI and yield. The dotted line box represents the optional input procedure of the mathematical regression for the ML or DNN regressor to the estimated LAI. The environmental data include solar radiation and maximum and minimum temperatures.


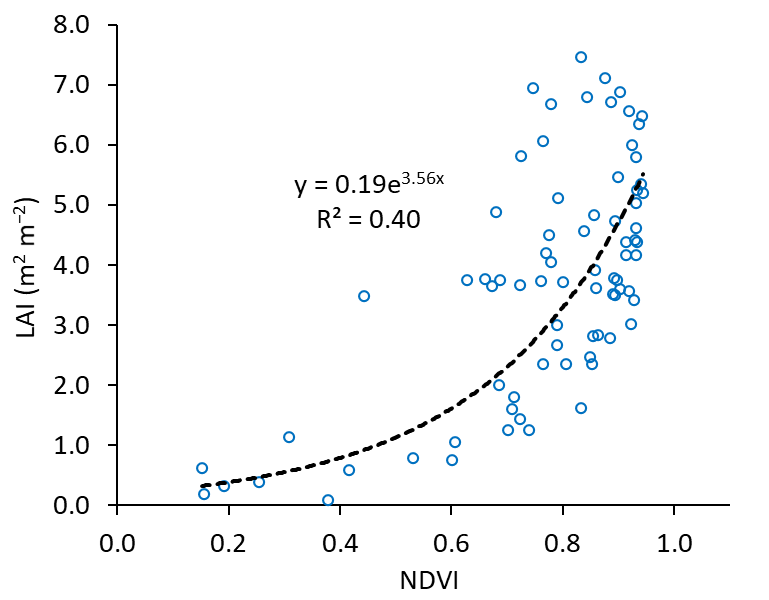


Supplementary Figure S2. Empirical relationship between LAI and NDVI of rice (n = 78).


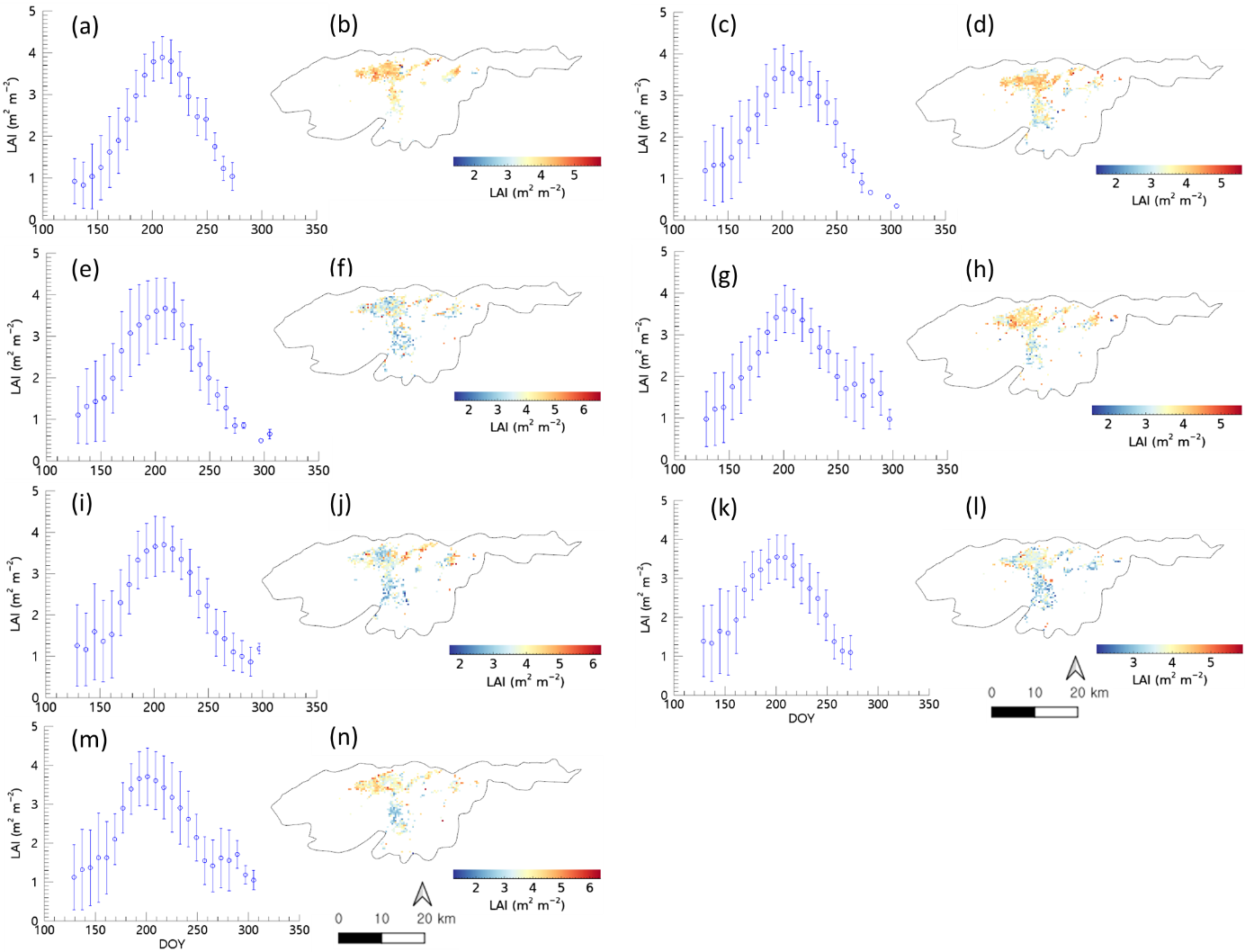


Supplementary Figure S3. Seasonal and geographical variations in LAI estimated from the MODIS-observed NDVI in Cheorwon, South Korea in (a and b) 2011, (c and d) 2012, (e and f) 2013, (g and h) 2014, (i and j) 2015, (k and l) 2016, and (m and n) 2017.


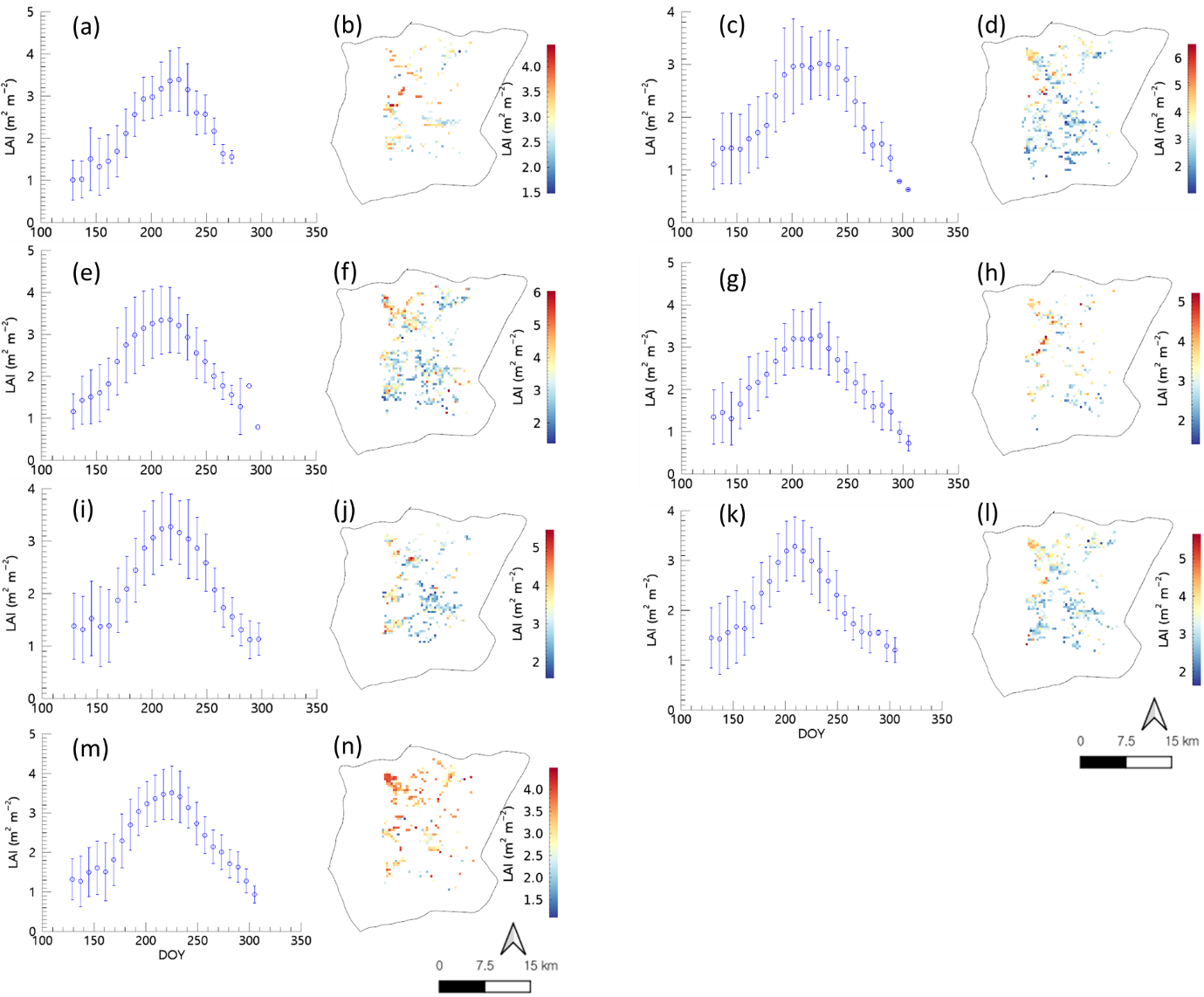


Supplementary Figure S4. Seasonal and geographical variations in LAI estimated from the MODIS-observed NDVI in Paju, South Korea in (a and b) 2011, (c and d) 2012, (e and f) 2013, (g and h) 2014, (i and j) 2015, (k and l) 2016, and (m and n) 2017.


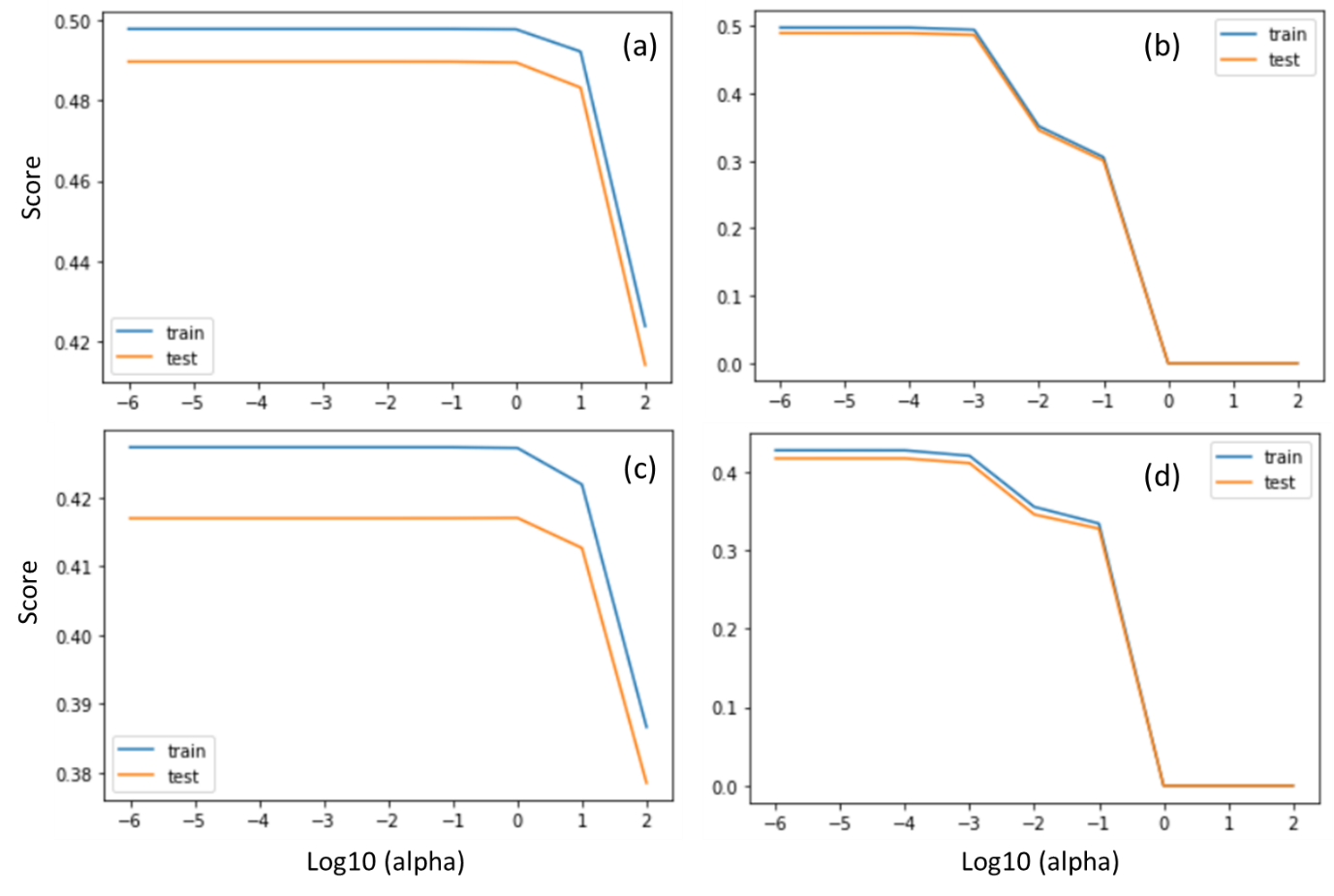


Supplementary Figure S5. Train and test scores of the Ridge (a and c) and Lasso (b and d) ML regressors for the Cheorwon (a and b) and Paju (c and d) datasets. The Alpha range used were 0.00001, 0.0001, 0.001, 0.01, 0.1, 0, 1, 100, and 1000.


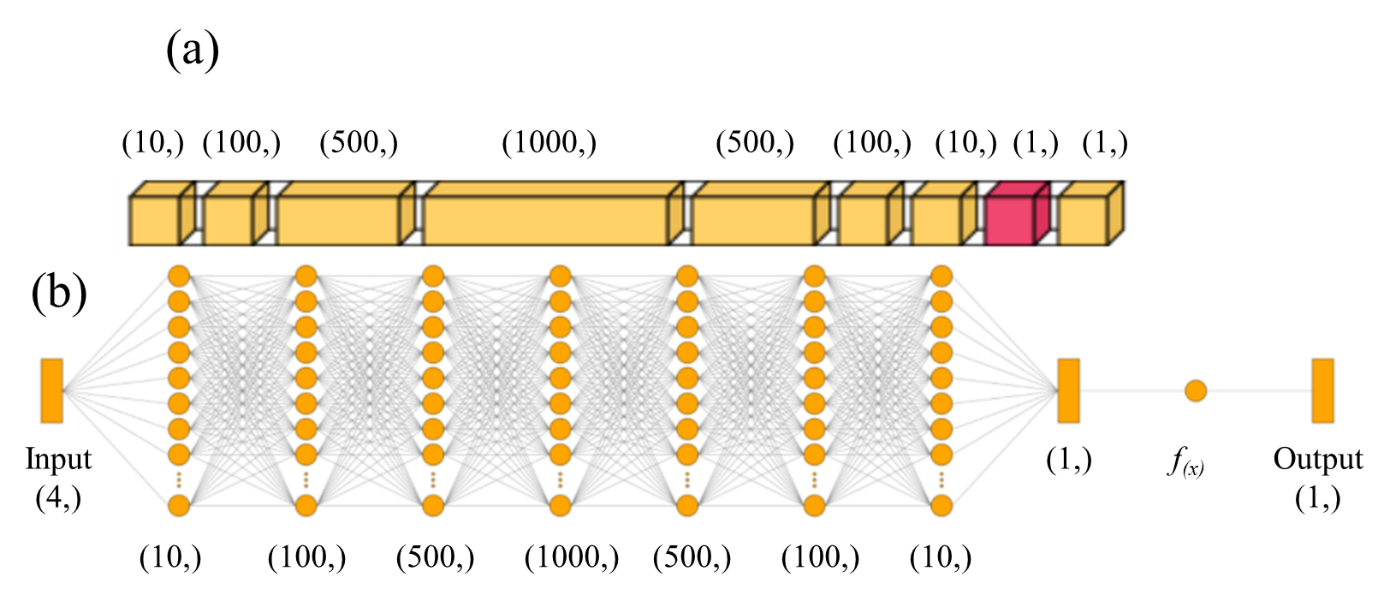


Supplementary Figure S6. Graphical representations of the DNN model: (a) the layered view and (b) the networked view. The numbers in the parentheses represent units in each layer, and *f*_(x)_ is the activation function.


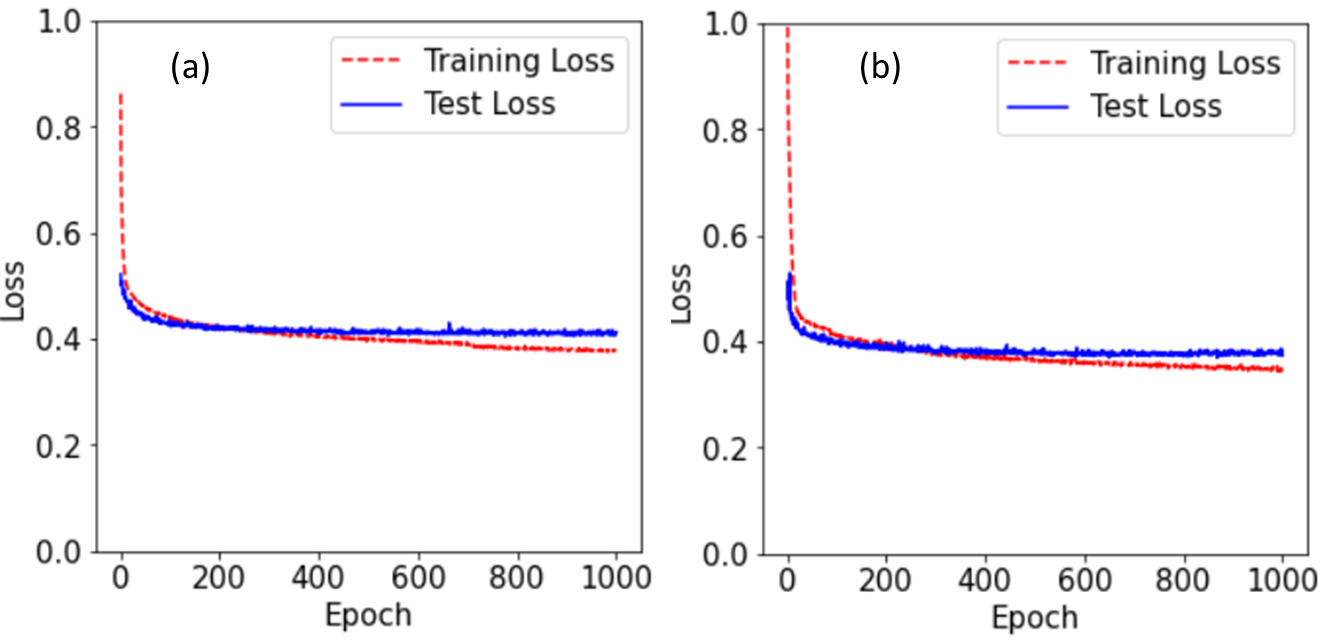


Supplementary Figure S7. Training and test losses of the DNN regressor for the Cheorwon (a) and Paju (b) datasets.
